# The prognostic differences and the effect of postmastectomy radiotherapy between post‐chemotherapy ypT1‐2ypN1 and de novo pT1‐2N1 breast cancer

**DOI:** 10.1002/cam4.5610

**Published:** 2023-02-03

**Authors:** Tian Yang, Xiaorong Zhong, Jun Wang, Zhongzheng Xiang, Yuanyuan Zeng, Siting Yu, Zelei Dai, Ningyue Xu, Ting Luo, Lei Liu

**Affiliations:** ^1^ Department of Head and Neck Oncology, Cancer Center, and State Key Laboratory of Biotherapy, West China Hospital Sichuan University Chengdu China; ^2^ Department of Radiation Oncology, Cancer Center, West China Hospital Sichuan University Chengdu China; ^3^ Breast Disease Center, Cancer Center, West China Hospital Sichuan University Chengdu China; ^4^ Multi‐omics Laboratory of Breast Diseases, State Key Laboratory of Biotherapy, National Collaborative, Innovation Center for Biotherapy, West China Hospital Sichuan University Chengdu China

**Keywords:** breast cancer, de novo pT1‐2N1, neoadjuvant chemotherapy, post‐chemotherapy ypT1‐2ypN1, postmastectomy radiotherapy, prognosis

## Abstract

**Background:**

The prognosis and the value of postmastectomy radiotherapy (PMRT) between post‐chemotherapy ypT1‐2ypN1 and de novo pT1‐2N1 breast cancer (BC) remain controversial. We aimed to evaluate the prognostic differences and the effect of PMRT between the two patient subsets.

**Methods:**

Patients diagnosed with pT1‐2N1M0 BC were identified between 2010 and 2018. The study endpoints were overall survival (OS), breast cancer‐specific survival (BCSS), locoregional recurrence‐free survival (LRFS), distant metastasis‐free survival (DMFS) and disease‐free survival (DFS). The chi‐square test, Kaplan–Meier method and Cox regression analysis were used for data analysis.

**Results:**

Total number of 2103 pT1‐2N1M0 BC patients were included in the study, including 270 post‐chemotherapy (97 without PMRT, 173 with PMRT) and 1833 de novo cases (993 without PMRT, 840 with PMRT). No significant differences were found between post‐chemotherapy ypT1‐2ypN1 and de novo pT1‐2N1 BC patients in 5‐year OS (*p* = 0.068), BCSS (*p* = 0.054), LRFS (*p* = 0.241), DMFS (*p* = 0.104) or DFS (*p* = 0.08). PMRT did not improve any survival outcome in patients receiving neoadjuvant chemotherapy; however, the PMRT group had a better 5‐year BCSS (97.0% vs. 95.8%, *p* = 0.033) in de novo pT1‐2N1 BC. Cox multivariate analysis demonstrated that PMRT was a significant independent predictor of BCSS (HR 0.628; 95% CI, 0.403–0.978; *p* = 0.04) in de novo pT1‐2N1 patients.

**Conclusions:**

There seemed no survival difference in post‐chemotherapy ypT1‐2ypN1 and de novo pT1‐2N1 BC patients with contemporary systemic therapy. In addition, PMRT might be exempted in patients with post‐chemotherapy ypT1‐2ypN1 BC, while not in patients with de novo pT1‐2N1 BC.

## Graphical abstract

Our study showed the long‐term outcomes regarding the combined intravitreal MTX and systemic HDMTX therapy in patients with vitreoretinal lymphoma (VRL). This combination approach induced a high survival rate with acceptable toxicities. Nevertheless, more than half of patients experienced CNS/systemic relapse, accounting for the main cause of death. Incorporation of more intensive consolidation strategies into the treatment protocol deserves to be considered, especially for patients with concurrent CNS disease at diagnosis.

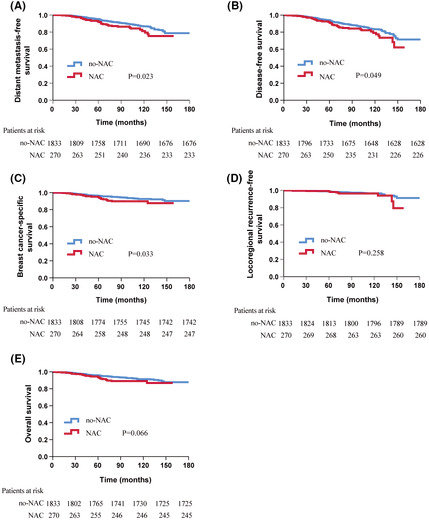

## 1 INTRODUCTION

Breast cancer (BC) has become the most frequently diagnosed cancer in female patients, with estimated 2.3 million (11.7% of all sites) new cases and 0.7 million (6.9% of all sites) new deaths worldwide in 2020.^1^ China accounts for approximately 18.3% of global newly diagnosed cases and 18% of female breast cancer‐related deaths all over the world.^2^ T1‐2N1 BC, a heterogeneous disease defined as tumor size ≤5 cm and 1–3 positive axillary lymph nodes, was well studied among all substages.

The value of chemotherapy for node‐positive BC patients has been well established.^3^ Neoadjuvant chemotherapy (NAC) can downstage primary tumors and axillary nodes, increase the eligibility for breast‐conserving surgery and improve the survival of patients with locally advanced BC.^4^, ^5^, ^6^, ^7^, ^8^, ^9^ However, it remains unclear whether there are the same survival and prognosis between patients with the same stages after NAC and at initial diagnosis, especially in pT1‐2N1M0 BC.^10^ Previous studies showed that the 5‐year LRFS, DMFS and OS rates of patients with de novo T1‐2N1 BC were 86.8%, 91.0%, and 83.8%, respectively,^11^ yet rare studies have explored the survival outcomes of post‐chemotherapy ypT1‐2ypN1 BC patients. Therefore, further studies should be conducted to solve the problem for better patient subset management.

It is still unclear whether there are the same treatment decisions between patients with the same pathological staging after or without NAC, and a similar dilemma exists in pT1‐2N1M0 BC regarding whether postmastectomy radiotherapy (PMRT) should be administered in this disease.^12^ The National Comprehensive Cancer Network (NCCN) Guidelines^13^ recommend that PMRT should be performed for patients with 1–3 positive axillary lymph nodes. The basis of the recommendation is derived from the result of the 2014 meta‐analysis published in Lancet that PMRT had significantly decreased loco‐regional recurrence (LRR) rate and cancer‐related death in patients with 1–3 involved lymph nodes.^14^ However, most of the trials enrolled in this meta‐analysis were completed before 1980 s, when the radiotherapy technique and chemotherapy regimens were far from what it is now, and the LRR rate for the patients without PMRT was up to 30% at that time.^15^, ^16^ Therefore, the value of PMRT in T1‐2N1 breast cancer should be revalidated in the era of contemporary systematic treatment.

In the modern systematic therapy era, there was no definite criteria for the prognostic differences and PMRT remains controversial for its indications in patients with pT1‐2N1M0 BC. Without consensus on whether all these patients would benefit from PMRT, the prognosis of post‐chemotherapy ypT1‐2ypN1 and de novo pT1‐2N1 BC patients are urgently needed to provide individualized precision therapy. Therefore, we sought to determine whether the survival outcomes and the impact of PMRT resemble on the two different groups of BC patients.

## 2 METHODS

### 2.1 Patients

BC patients treated at West China Hospital of Sichuan University were enrolled in our study. Inclusion criteria: (i) diagnosed between 2008 and 2018; (ii) pathological T1‐2N1M0 breast cancer; (iii) receiving mastectomy; (iv) with adjuvant chemotherapy and (v) having sufficient clinicopathologic and follow‐up data. We excluded male patients, patients with bilateral and metastatic BC. The protocols were approved by the Ethical Review Board of West China Hospital of Sichuan University (Approval number: 2020427). Written informed consent was obtained from all individual participants included in the study.

### 2.2 Treatments and endpoints

All patients were treated with mastectomy and adjuvant chemotherapy. High‐risk patients (such as advanced T stage, advanced *N* stage, and triple negative breast cancer) received additional NAC, while de novo patients only received adjuvant chemotherapy regularly. In adjuvant chemotherapy setting, 2036 patients (96.8%) received a taxane or anthracycline‐based chemotherapy regimen, and 67 patients (3.2%) received other chemotherapy regimens, such as cyclophosphamide, methotrexate and 5‐fluorouracil (CMF) regimen and oral chemotherapy drugs. For NAC treatment, 260 patients (96.3%) received a taxane or anthracycline‐based chemotherapy, and 10 patients (3.7%) received other chemotherapy regimens. PMRT was administered with a total dose of 40–50 gray, divided into 12–30 fractions. In addition, 1576 patients (accounted for 74.9% of all BC patients) with positive estrogen receptor (ER) or progesterone receptor (PR) received endocrine therapy, and 290 patients (accounted for 13.8% of all BC patients) with positive human epidermal growth factor receptor 2 (HER2) received targeted therapy.

The endpoints were defined as follows: LRFS, defined as no relapse in the locoregional region; DMFS, defined as no relapse outside the locoregional region during the observation period; OS, defined as no death during the first diagnosis to the last follow‐up; BCSS, defined as no death from breast cancer during observation and DFS, defined as no treatment failure or death from any cause.

### 2.3 Statistics

The Chi‐square test was used to compare the clinicopathologic characteristics between patients who received PMRT and those who did not. The survival curves for LRFS, DMFS, BCSS, DFS and OS were portrayed using the Kaplan–Meier method, and the log‐rank test was used to compare the survival differences. A Cox regression hazards model was used to identify the independent risk factor for patients receiving neoadjuvant chemotherapy or PMRT. A *p* < 0.05 (two‐sided) was considered significant. All statistical analyses were performed using SPSS 24.0 (SPSS Inc.).

## 3 RESULTS

### 3.1 Patient characteristics

The baseline characteristics of 2103 eligible patients were summarized in Table 1. The median age of the patients was 48 years (range 23–77) and 57.8% of whom were younger than 50 years old. Among all the patients, 1263 (60.1%) were premenopausal and the rest 840 (39.9%) were postmenopausal. Most patients had pathologic infiltrating ductal carcinoma (94.9%), poorly differentiated (60.3%), T2 (68.0%), ER positive (74.8%), PR positive (68.6%) and HER2 negative (74.2%) disease.

**TABLE 1:** Clinicopathologic characteristics of patients with pT1‐2N1M0 breast cancer.

		NAC			no‐NAC		
Variables	*N* (%)	PMRT	no‐PMRT	*p* value	PMRT	no‐PMRT	*p* value
Age (years)				**0.046**			**<0.001**
<50	1215 (57.8)	114 (65.9)	52 (53.6)		532 (63.3)	517 (52.1)	
≥50	888 (42.2)	59 (34.1)	45 (46.4)		308 (36.7)	476 (47.9)	
Menstrual state				0.09			**<0.001**
Premenstrual	1263 (60.1)	116 (67.1)	55 (56.7)		562 (66.9)	530 (53.4)	
Postmenstrual	840 (39.9)	57 (32.9)	42 (43.3)		278 (33.1)	463 (46.6)	
Pathological type				0.464			0.893
Infiltrating ductal carcinoma	1996 (94.9)	156 (90.2)	88 (90.7)		801 (95.4)	951 (95.8)	
Lobular carcinoma	25 (1.2)	1 (0.6)	2 (2.1)		11 (1.3)	11 (1.1)	
Other	82 (3.9)	16 (9.2)	7 (7.2)		28 (3.3)	31 (3.1)	
Grade				0.763			0.255
Well‐differentiated	51 (2.4)	2 (1.2)	2 (2.1)		18 (2.1)	29 (2.9)	
Moderately differentiated	784 (37.3)	40 (23.1)	20 (20.6)		320 (38.1)	404 (40.7)	
Poorly differentiated/undifferentiated	1268 (60.3)	131 (75.7)	75 (77.3)		502 (59.8)	560 (56.4)	
T stage				0.877			0.073
T1	674 (32.0)	28 (16.2)	15 (14.5)		271 (32.3)	360 (36.3)	
T2	1429 (68.0)	145 (83.8)	82 (84.5)		569 (67.7)	633 (63.7)	
The 7th AJCC staging				0.877			0.073
IIA	674 (32.0)	28 (16.2)	15 (14.5)		271 (32.3)	360 (36.3)	
IIB	1429 (68.0)	145 (83.8)	82 (84.5)		569 (67.7)	633 (63.7)	
ER				0.272			0.134
Negative	530 (25.2)	58 (33.5)	39 (40.2)		212 (25.2)	221 (22.3)	
Positive	1573 (74.8)	115 (66.5)	58 (59.8)		628 (74.8)	772 (77.7)	
PR				0.448			0.549
Negative	660 (31.4)	72 (41.6)	45 (46.4)		243 (28.9)	300 (30.2)	
Positive	1443 (68.6)	101 (58.4)	52 (53.6)		597 (71.1)	693 (69.8)	
HER2				0.652			**0.004**
Negative	1560 (74.2)	124 (71.7)	67 (69.1)		601 (71.5)	768 (77.3)	
Positive	543 (25.8)	49 (28.3)	30 (30.9)		239 (28.5)	225 (22.7)	
Endocrine therapy				**<0.001**			<0.105
No	527 (25.1)	38 (22.0)	42 (43.3)		190 (22.6)	257 (25.9)	
Yes	1576 (74.9)	135 (78.0)	55 (56.7)		650 (77.4)	736 (74.1)	
Targeted therapy				0.15			**0.004**
No	1813 (86.2)	152 (87.9)	79 (81.4)		704 (83.8)	878 (88.4)	
Yes	290 (13.8)	21 (12.1)	18 (18.6)		136 (16.2)	115 (11.6)	

Abbreviations: AJCC, American Joint Committee on Cancer; ER, estrogen receptor; HER2, human epidermal growth factor receptor 2; NAC, neoadjuvant chemotherapy; PMRT, postmastectomy radiotherapy; PR, progesterone receptor.

Bold values represent the statistically significant (*p* < 0.05).

In total, 12.8% (*n* = 270) of the patients received NAC and 48.1% (*n* = 1013) received PMRT. In the post‐chemotherapy ypT1‐2ypN1 group, 64.1% (*n* = 173) patients received PMRT and patients younger than 50 years (*p* = 0.046) were more likely to receive PMRT. In the de novo pT1‐2N1 group, PMRT was administered in 45.8% (*n* = 840) of the patients. Younger than 50 years (*p* < 0.001), premenopausal (*p* < 0.001) and HER2 overexpression (*p* = 0.004) were associated with higher rate of PMRT. Adjuvant endocrine therapy was administered in 1576 (accounted for 94.1% of all patients) hormone receptor positive patients, and anti‐HER2 therapy was conducted in 290 (accounted for 86.2% of all patients) HER2‐positive patients.

### 3.2 Prognostic differences in post‐chemotherapy ypT1‐2ypN1 and de novo pT1‐2N1 BC patients

**FIGURE 1:**
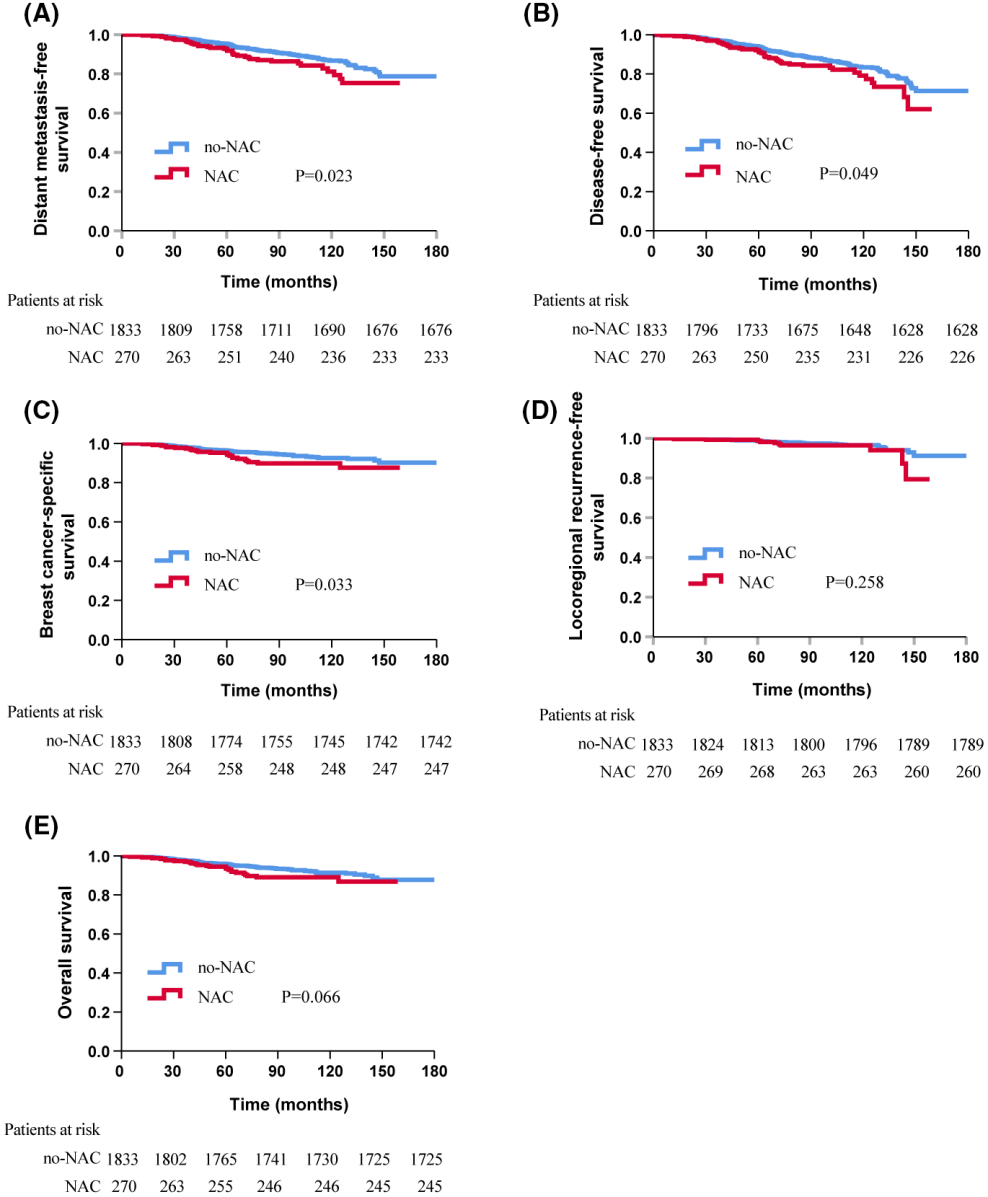
Kaplan–Meier survival curve of survival outcomes in c for the entire cohort. (A) distant metastasis‐free survival; (B) disease‐free survival; (C) breast cancer‐specific survival; (D) locoregional recurrence‐free survival; (E) overall survival.

With a median follow‐up of 76.0 months (range 0.1–252.6 months), 55 (2.6%) local recurrences, 194 (9.2%) distant metastases, 133 (6.3%) deaths, and 114 (5.4%) breast cancer‐related deaths occurred. Survival analysis (Figure 1) showed that the de novo pT1‐2N1 group had better 5‐year DMFS (95.3% vs. 92.8%, *p* = 0.023), DFS (93.8% vs. 92.1%, *p* = 0.049) and BCSS (96.4% vs. 95.3%, *p* = 0.033) than the post‐chemotherapy ypT1‐2ypN1 group in the entire cohort. However, there were no significant differences in 5‐year LRFS (99.2% vs. 98.7%, *p* = 0.258) or OS (94.5% vs. 95.9%, *p* = 0.066) between the two groups. Multivariate Cox regression analysis (Tables [Link], [Link]) showed that NAC was not a significant risk factor for OS (hazard ratio, HR 1.512; 95% CI, 0.970–2.356; *p* = 0.068), BCSS (HR 1.584; 95% CI, 0.993–2.529; *p* = 0.054), LRFS (HR 1.52; 95% CI, 0.755–3.063; *p* = 0.241), DMFS (HR 1.358; 95% CI, 0.939–1.964; *p* = 0.104) or DFS (HR 1.347; 95% CI, 0.965–1.879; *p* = 0.08) in pT1‐2N1M0 BC patients.

### 3.3 Impact of PMRT in post‐chemotherapy ypT1‐2ypN1 and de novo pT1‐2N1 BC patients

For the pT1‐2N1M0 BC patients in the whole cohort, PMRT was significantly correlated with better 5‐year OS (96.6% vs. 94.8%, *p* = 0.003) and BCSS (96.9% vs 95.6%, *p* = 0.010), while no sign of improvement in the 5‐year LRFS (99.0% vs. 98.6%, *p* = 0.091), DMFS (94.8% vs. 95.1%, *p* = 0.542) or DFS (94.0% vs. 93.1%, *p* = 0.054) were observed (Supplementary Figure S1). Multivariate Cox regression analysis (Tables [Link], [Link]) revealed that PMRT was the only significant factor associated with increased OS (HR 0.608; 95% CI, 0.421–0.877; *p* = 0.008), BCSS (HR 0.585; 95% CI, 0.395–0.869; *p* = 0.008), LRFS (HR 0.532; 95% CI, 0.300–0.945; *p* = 0.031) and DFS (HR 0.76; 95% CI, 0.586–0.986; *p* = 0.039) except for DMFS (HR 0.849; 95% CI, 0.633–1.137; *p* = 0.272) within all pT1‐2N1M0 BC patients.

**FIGURE 2:**
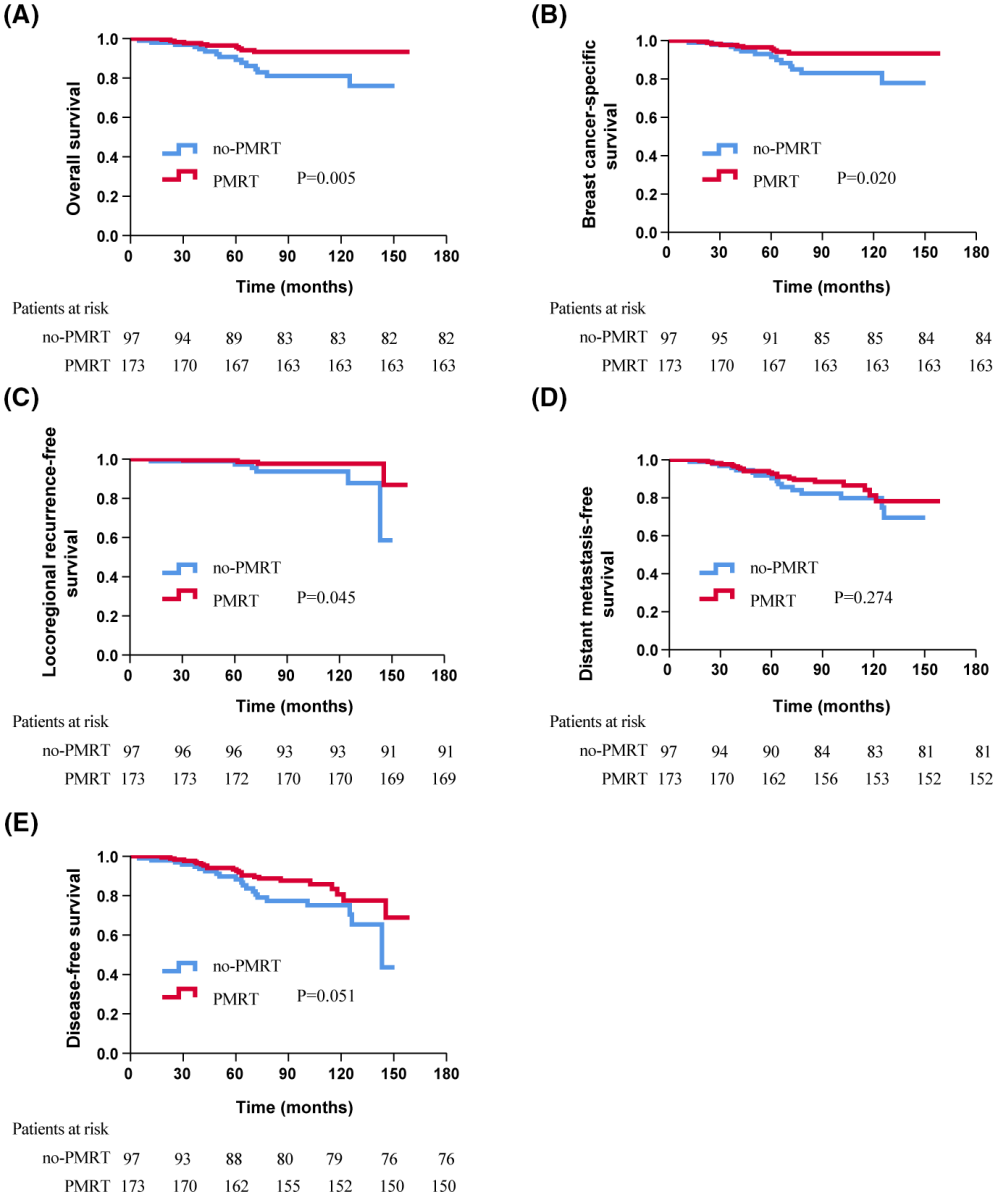
Kaplan–Meier survival curve of survival outcomes in post‐chemotherapy ypT1‐2ypN1 BC patients with or without PMRT. (A) overall survival; (B) breast cancer‐specific survival; (C) locoregional recurrence‐free survival; (D) distant metastasis‐free survival; (E) disease‐free survival.

For the post‐chemotherapy ypT1‐2ypN1 subgroup, BC patients who received PMRT had superior 5‐year OS (96.4% vs. 90.7%, *p* = 0.005), BCSS (96.4% vs. 93.0%, *p* = 0.020) and LRFS (99.4% vs. 98.9%, *p* = 0.045). However, the benefit of PMRT was not statistically significant in DMFS (93.3% vs. 91.8%, *p* = 0.274) or DFS (93.3% vs. 89.7%, *p* = 0.051, Figure 2). PMRT was not identified to be an independent factor of LRFS (HR 0.429; 95% CI, 0.097–1.898; *p* = 0.265), DFS (HR 0.781; 95% CI, 0.413–1.477; *p* = 0.448), DMFS (HR 0.826; 95% CI, 0.416–1.641; *p* = 0.585), OS (HR 0.555; 95% CI, 0.227–1.354; *p* = 0.195) or BCSS (HR 0.585; 95% CI, 0.237–1.443; *p* = 0.224) in post‐chemotherapy ypT1‐2ypN1 BC patients from the multivariate analysis (Table 2).

**TABLE 2:** Multivariate Cox regression analysis of survival outcomes in post‐chemotherapy ypT1‐2ypN1 and de novo pT1‐2N1 BC patients.

	post‐chemotherapy			de novo		
	HR	95% CI	*p*	HR	95% CI	*p*
LRFS						
no‐PMRT	1			1		
PMRT	0.429	0.097–1.898	0.265	0.613	0.326–1.153	0.129
DFS						
no‐PMRT	1			1		
PMRT	0.781	0.413–1.477	0.448	0.796	0.597–1.060	0.119
DMFS						
no‐PMRT	1			1		
PMRT	0.826	0.416–1.641	0.585	0.894	0.645–1.239	0.501
OS						
no‐PMRT	1			1		
PMRT	0.555	0.227–1.354	0.195	0.668	0.445–1.003	0.052
BCSS						
no‐PMRT	1			1		
PMRT	0.585	0.237–1.443	0.224	0.628	0.403–0.978	**0.04**

Abbreviations: BCSS, breast cancer‐specific survival; CI, confidence interval; DFS, disease‐free survival; DMFS, distant metastasis‐free survival; HR, Hazard Ratio; LRFS, recurrence‐free survival; OS, overall survival; PMRT, postmastectomy radiotherapy.

Bold values represent the statistically significant (*p* < 0.05).

**FIGURE 3:**
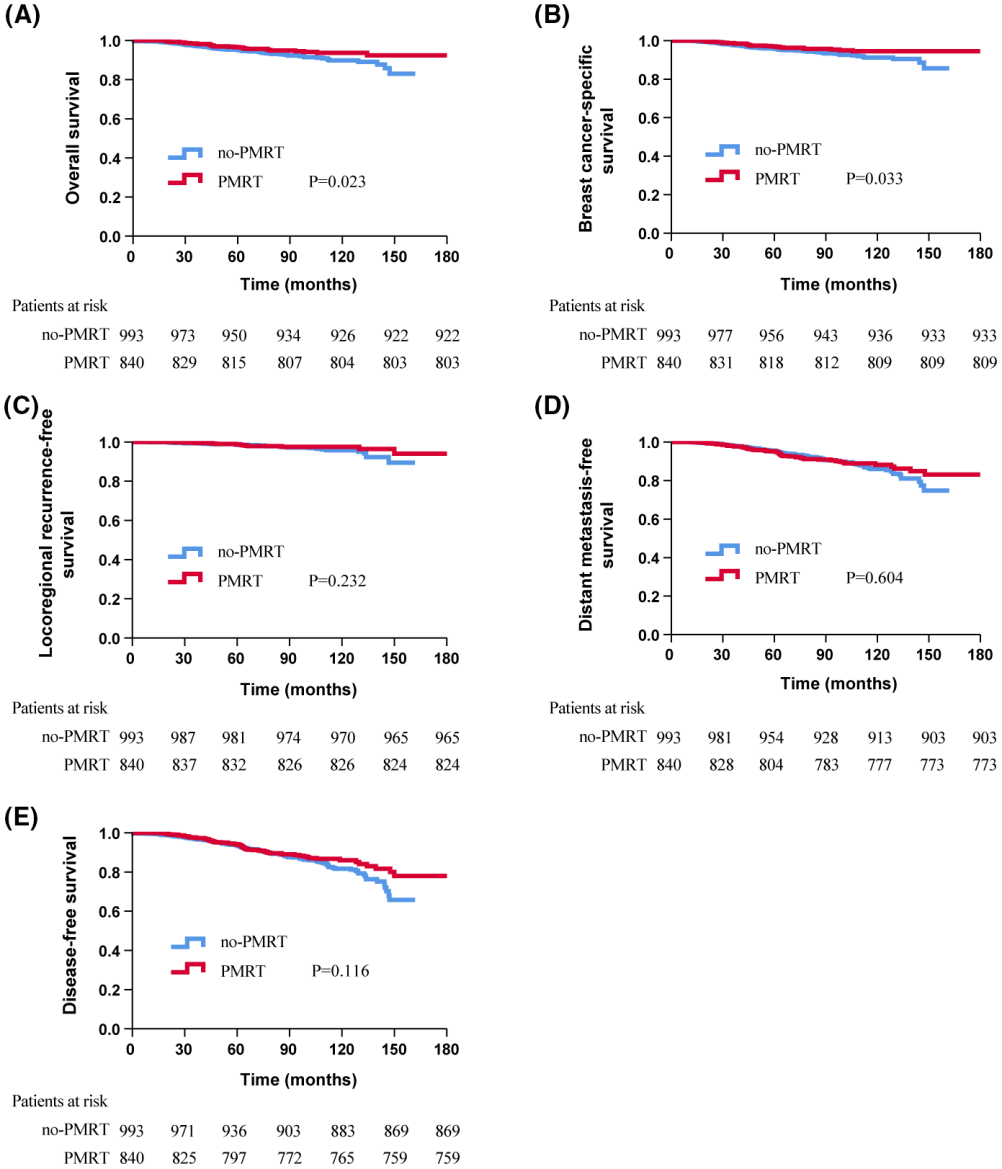
Kaplan–Meier survival curve of c with or without PMRT. (A) overall survival; (B) breast cancer‐specific survival; (C) locoregional recurrence‐free survival; (D) distant metastasis‐free survival; (E) disease‐free survival.

For the de novo pT1‐2N1 subgroup, BC patients derived a significant benefit from PMRT in 5‐year OS (96.7% vs. 95.2%, *p* = 0.023) and BCSS (97.0% vs. 95.8%, *p* = 0.033). However, no benefit from PMRT was observed in LRFS (98.9% vs 98.6%, *p* = 0.232), DMFS (95.1% vs. 95.4%, *p* = 0.604) or DFS (94.2% vs. 93.4%, *p* = 0.116, Figure 3). On multivariate analysis (Table 2) among the patients without NAC, PMRT remained a statistically significant predictor of BCSS (HR 0.628; 95% CI, 0.403–0.978; *p* = 0.04), while it failed to bring better outcomes in LRFS (HR 0.613; 95% CI, 0.326–1.153; *p* = 0.129), DFS (HR 0.796; 95% CI, 0.597–1.060; *p* = 0.119), DMFS (HR 0.894; 95% CI, 0.645–1.239; *p* = 0.501) or OS (HR 0.668; 95% CI, 0.445–1.003; *p* = 0.052).

## 4 DISCUSSION

The aim of our study was to validate the different prognosis and the impact of PMRT between post‐chemotherapy ypT1‐2ypN1 and de novo pT1‐2N1 BC patients. Our results demonstrated that there were no significant prognostic differences in OS, BCSS, LRFS, DMFS or DFS between the two groups. PMRT was associated with a better survival outcome in the entire cohort and de novo pT1‐2N1 patients, while not in post‐chemotherapy ypT1‐2ypN1 patients.

The value of NAC in downstaging primary tumors in BC patients has been extensively studied and patients with locally advanced BC achieved better survival outcomes after NAC.^17^, ^18^ However, the prognostic differences of de novo and post‐chemotherapy BC patients with the same pathological stages remain controversial, especially in pT1‐2N1M0. Some scholars proposed that good survival outcomes were found in post‐chemotherapy BC patients, and Wang et al. demonstrated that the 5‐year LRFS, DMFS, DFS and OS rates of post‐chemotherapy BC patients were 88%, 79%, 73%, and 81%, respectively, but rare studies focused on the prognosis of patients with post‐chemotherapy ypT1‐2ypN1.^19^, ^20^, ^21^ Previous studies have been devoted to exploring the survival of early‐stage de novo BC patients.^22^, ^23^ A study from Shen et al. argued that the five‐year LRFS, DMFS and OS rates of de novo T1‐2N1 BC patients were 86.8%, 91.0% and 83.8%, respectively.^11^ However, the survival differences between post‐chemotherapy ypT1‐2ypN1 and de novo pT1‐2N1 BC patients have not been reported thus far. Therefore, our study first revealed that, there were no significant differences in prognosis between these two groups with modern systemic therapy, regardless of OS, BCSS, LRFS, DMFS or DFS. Post‐chemotherapy ypT1‐2ypN1 BC patients might have worse initial clinical TN stages or pathologic responses compared to de novo pT1‐2N1 BC patients, but we found no significant prognostic differences in these two populations, possibly because of the better prognosis of post‐chemotherapy BC patients with new NAC regimens such as adriamycin and taxol in modern treatment modalities.

The role of PMRT in whole pT1‐2N1M0 BC patients and the heterogenous impact of PMRT in patient subsets remain controversial.^24^ Previous studies demonstrated that PMRT had no effect on prognosis in pT1‐2N1M0 BC patients,^25^, ^26^ but other studies concluded that PMRT was valuable.^27^, ^28^, ^29^ The EBCTCG meta‐analysis clarified the benefit of PMRT in node‐positive BC patients and confirmed that radiotherapy reduced locoregional recurrence, overall recurrence and BC mortality in 1314 patients with 1–3 positive nodes.^30^ However, most of these studies dated back to 30 years ago,^31^ which may not be suitable for BC patients in the modern era, and the role of PMRT was not further analyzed in patient subgroups. Consistent with the findings of these studies, we observed that PMRT could improve the survival outcomes of pT1‐2N1M0 BC patients in OS and BCSS in the entire cohort, however, we hypothesized that the prognostic impact of PMRT may differ in different subgroups.

Previous studies evaluated the effects of PMRT on post‐chemotherapy and de novo BC patients.^32^, ^33^, ^34^ Some researchers believed that BC patients with ypN1 did not benefit from PMRT, but there was a small number of patients and a lack of unified T staging in these studies.^6^, ^32^, ^35^ Zhang et al. reported that whole breast radiation therapy improved OS, LRFS and DMFS in ypN0‐3 BC patients with invasive ductal carcinoma, but the study included BC patients who received breast‐conserving surgery and systemic therapy preoperatively.^36^ A retrospective study confirmed that PMRT had no benefits on the OS and progression‐free survival of de novo T1‐2N1 BC patients, but the number of the patients was relatively small.^28^ Luo et al. suggested that PMRT was not associated with the breast cancer mortality (BCM) in older T1‐2N1 BC patients who received chemotherapy, but decreased the BCM in those who had no chemotherapy.^37^ However, the study focused on seniors (aged 70 years or older) only and it did not specify whether those patients received NAC or adjuvant chemotherapy. Our research focused on post‐mastectomy pT1‐2N1M0 BC patients and presented that PMRT had a positive influence on OS and BCSS in the whole cohort, however, in subgroups, it could only improve the BCSS in de novo pT1‐2N1 BC patients and may be omitted in post‐chemotherapy ypT1‐2ypN1 BC patients due to no beneficial prognosis in OS, DMFS, LRFS, BCSS or DFS. We only observed the benefits of PMRT in de novo pT1‐2N1 BC patients, there may be the following reasons, most of the previous chemotherapy regimens were primarily based on CMF, but BC patients received modern NAC regimens in our current cohort, including adriamycin and taxol, and the excellent effects of modern chemotherapy might exceed the benefits of PMRT in post‐chemotherapy ypT1‐2ypN1 BC patients.

Our retrospective study still has several limitations. First, it is a single institution study with a heterogeneous cohort, and more multi‐institutional studies are needed for better conclusive interpretation of the results. Furthermore, potential selection bias might have existed due to its retrospective nature and two homogeneous study groups were classified to compensate for this. Finally, our database lacks the information of initial clinical staging in patients receiving NAC and the limited follow‐up time might underestimate the actual survival benefit of PMRT for patients, therefore, further prospective studies are required to confirm our results with a longer follow‐up period. Nevertheless, this cohort reflects a real‐world experience and it was the first study to investigate the prognostic differences and the role of PMRT in post‐chemotherapy ypT1‐2ypN1 and de novo pT1‐2N1 BC patients with a large sample size. Our study answered the question whether post‐chemotherapy ypT1‐2ypN1 and de novo pT1‐2 N1 patients had the same prognosis in the situation of not knowing the initial clinical stage of patients receiving NAC. Therefore, our results might provide guidance for the management of pT1‐2N1M0 BC patients.

## 5 CONCLUSIONS

In conclusion, our study demonstrated that there were no significant prognostic differences in post‐chemotherapy ypT1‐2ypN1 and de novo pT1‐2N1 BC patients with modern systemic therapy. PMRT benefitted de novo pT1‐2N1 BC patients in BCSS, conversely, it might be omitted in BC patients with post‐chemotherapy ypT1‐2ypN1. This retrospective study helps to determine the necessity of performing PMRT in pT1‐2N1M0 BC patients.

## AUTHOR CONTRIBUTIONS

**Tian Yang:** Conceptualization (lead); formal analysis (equal); investigation (equal); methodology (lead); writing – original draft (lead). **Xiaorong Zhong:** Formal analysis (equal); investigation (lead); methodology (equal); writing – original draft (equal). **Jun Wang:** Formal analysis (lead); investigation (equal); methodology (equal); writing – original draft (equal). **Zhongzheng Xiang:** Formal analysis (equal); methodology (equal). **Yuan‐yuan Zeng:** Formal analysis (equal); investigation (equal). **Siting Yu:** Methodology (equal); visualization (equal). **Zelei Dai:** Conceptualization (equal); supervision (equal). **Ningyue Xu:** Conceptualization (equal); supervision (equal). **Ting Luo:** Supervision (equal); validation (equal); writing – review and editing (equal). **Lei Liu:** Supervision (equal); visualization (equal); writing – review and editing (lead).

## FUNDING INFORMATION

The study was supported by the project of Science and Technology Department of Sichuan Province (23NSFSC0825).

## CONFLICT OF INTEREST STATEMENT

The authors declare no conflict of interest.

## Supporting information


Figure S1.

Table S1

Table S2.

## Data Availability

The data that support the findings of this study are available from the corresponding author upon reasonable request.
