# Supplementary figures and images for: The prognostic differences and the effect of postmastectomy radiotherapy between post‐chemotherapy ypT1‐2ypN1 and de novo pT1‐2N1 breast cancer
Source: Cancer Med. 2023 Feb 3;12(7):8112–21. doi: 10.1002/cam4.5610 (PMC10134268; doi:10.1002/cam4.5610)

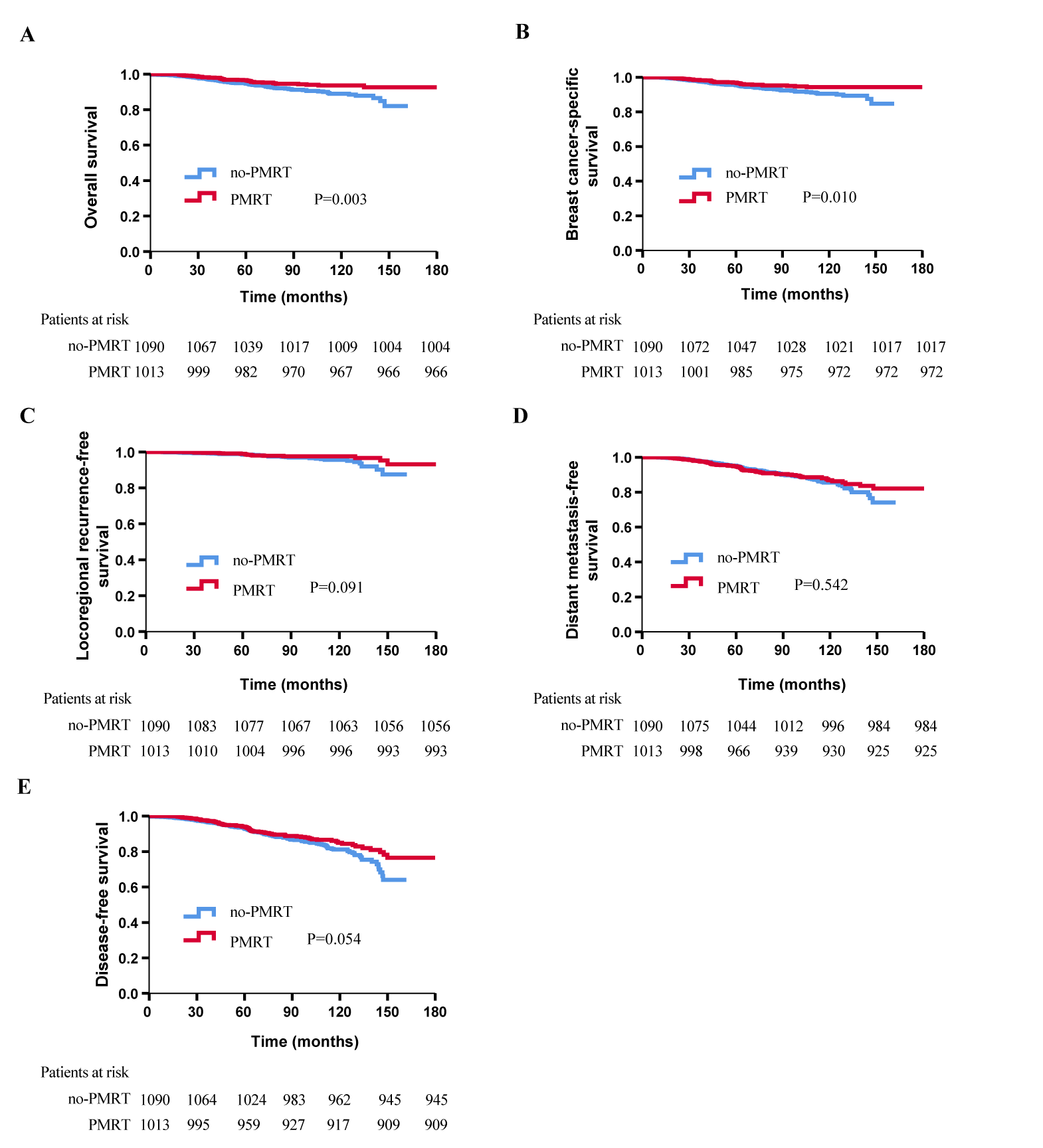

Supplement: Supplementary file 1 — Figure S1. [file CAM4-12-8112-s001.tif]
