# Supplementary material for: The prognostic differences and the effect of postmastectomy radiotherapy between post‐chemotherapy ypT1‐2ypN1 and de novo pT1‐2N1 breast cancer
Source: Cancer Med. 2023 Feb 3;12(7):8112–21. doi: 10.1002/cam4.5610 (PMC10134268; doi:10.1002/cam4.5610)
Supplement: Supplementary file 2 — Table S1 [file CAM4-12-8112-s003.docx]

Supplementary Table 1. Multivariate Cox regression analysis of prognostic factors for outcomes of OS and BCSS in pT1-2N1M0 BC patients

| Variables | OS |  |  | BCSS |  |  |
| --- | --- | --- | --- | --- | --- | --- |
|  | HR | 95% CI | P | HR | 95% CI | P |
| Age (years) |  |  |  |  |  |  |
| <50 | 1 |  |  | 1 |  |  |
| ≥50 | 0.809 | 0.466-1.403 | 0.450 | 0.87 | 0.477-1.586 | 0.649 |
| Menstrual state |  |  |  |  |  |  |
| Premenstrual | 1 |  |  | 1 |  |  |
| Postmenstrual | 1.868 | 1.077-3.240 | **0.026** | 1.543 | 0.848-2.807 | 0.156 |
| Pathological type |  |  |  |  |  |  |
| Infiltrating ductal carcinoma | 1 |  |  | 1 |  |  |
| Lobular carcinoma | 1.758 | 0.428-7.228 | 0.434 | 1.874 | 0.454-7.742 | 0.385 |
| Other | 0.587 | 0.186-1.858 | 0.365 | 0.634 | 0.200-2.013 | 0.44 |
| Grade |  |  |  |  |  |  |
| Well differentiated | 1 |  |  | 1 |  |  |
| Moderately differentiated | 1.039 | 0.246-4.390 | 0.958 | 1.508 | 0.202-11.262 | 0.689 |
| Poorly differentiated/undifferentiated | 1.840 | 0.447-7.584 | 0.398 | 3.134 | 0.431-22.777 | 0.259 |
| T stage |  |  |  |  |  |  |
| T1 | 1 |  |  | 1 |  |  |
| T2 | 1.432 | 0.952-2.155 | 0.085 | 1.597 | 1.012-2.520 | **0.044** |
| ER |  |  |  |  |  |  |
| Negative | 1 |  |  |  |  |  |
| Positive | 1.437 | 0.758-2.722 | 0.267 | 1.227 | 0.613-2.454 | 0.563 |
| PR |  |  |  |  |  |  |
| Negative | 1 |  |  | 1 |  |  |
| Positive | 0.862 | 0.525-1.417 | 0.558 | 0.758 | 0.448-1.282 | 0.302 |
| HER2 |  |  |  |  |  |  |
| Negative | 1 |  |  | 1 |  |  |
| Positive | 0.533 | 0.33-0.860 | **0.01** | 0.55 | 0.33-0.917 | **0.022** |
| Neoadjuvant chemotherapy |  |  |  |  |  |  |
| No | 1 |  |  | 1 |  |  |
| Yes | 1.512 | 0.970-2.356 | 0.068 | 1.584 | 0.993-2.529 | 0.054 |
| Endocrine therapy |  |  |  |  |  |  |
| No | 1 |  |  | 1 |  |  |
| Yes | 0.494 | 0.276-0.885 | **0.018** | 0.702 | 0.366-1.347 | 0.288 |
| Targeted therapy |  |  |  |  |  |  |
| No | 1 |  |  | 1 |  |  |
| Yes | 2.874 | 1.737-4.756 | **<0.001** | 3.396 | 2.009-5.740 | **<0.001** |
| PMRT |  |  |  |  |  |  |
| No | 1 |  |  | 1 |  |  |
| Yes | 0.608 | 0.421-0.877 | **0.008** | 0.585 | 0.395-0.869 | **0.008** |

OS, overall survival; BCSS, breast cancer-specific survival; HR, Hazard Ratio; CI, confidence interval; PMRT, postmastectomy radiotherapy; AJCC, American Joint Committee on Cancer; ER, estrogen receptor; PR, progesterone receptor; HER2, human epidermal growth factor receptor 2.
