# Supplementary material for: The prognostic differences and the effect of postmastectomy radiotherapy between post‐chemotherapy ypT1‐2ypN1 and de novo pT1‐2N1 breast cancer
Source: Cancer Med. 2023 Feb 3;12(7):8112–21. doi: 10.1002/cam4.5610 (PMC10134268; doi:10.1002/cam4.5610)
Supplement: Supplementary file 3 — Table S2. [file CAM4-12-8112-s002.docx]

Supplementary Table 2. Multivariate Cox regression analysis of prognostic factors for outcomes of LRFS, DMFS and DFS in pT1-2N1M0 BC patients

| Variables | LRFS |  |  | DMFS |  |  | DFS |  |  |
| --- | --- | --- | --- | --- | --- | --- | --- | --- | --- |
|  | HR | 95% CI | P | HR | 95% CI | P | HR | 95% CI | P |
| Age (years) |  |  |  |  |  |  |  |  |  |
| <50 | 1 |  |  | 1 |  |  | 1 |  |  |
| ≥50 | 0.8 | 0.329-1.941 | 0.621 | 0.926 | 0.588-1.458 | 0.740 | 0.846 | 0.568-1.262 | 0.413 |
| Menstrual state |  |  |  |  |  |  |  |  |  |
| Premenstrual | 1 |  |  | 1 |  |  | 1 |  |  |
| Postmenstrual | 1.253 | 0.524-2.997 | 0.613 | 1.594 | 1.017-2.497 | **0.042** | 1.774 | 1.193-2.639 | **0.005** |
| Pathological type |  |  |  |  |  |  |  |  |  |
| Infiltrating ductal carcinoma | 1 |  |  | 1 |  |  | 1 |  |  |
| Lobular carcinoma | 0 | 0-3.901 | 0.97 | 2.073 | 0.652-6.592 | 0.217 | 2.204 | 0.809-6.003 | 0.122 |
| Other | 0.469 | 0.064-3.436 | 0.456 | 0.935 | 0.436-2.004 | 0.862 | 0.892 | 0.438-1.815 | 0.752 |
| Grade |  |  |  |  |  |  |  |  |  |
| Well differentiated | 1 |  |  | 1 |  |  | 1 |  |  |
| Moderately differentiated | 0.625 | 0.078-4.975 | 0.657 | 0.976 | 0.301-3.160 | 0.967 | 1.067 | 0.386-2.949 | 0.9 |
| Poorly differentiated/undifferentiated | 1.193 | 0.16-8.906 | 0.863 | 1.383 | 0.435-4.390 | 0.583 | 1.426 | 0.524-3.886 | 0.487 |
| T stage |  |  |  |  |  |  |  |  |  |
| T1 | 1 |  |  | 1 |  |  | 1 |  |  |
| T2 | 1.58 | 0.823-3.034 | 0.17 | 1.998 | 1.390-2.872 | **<0.001** | 1.658 | 1.221-2.251 | **0.001** |
| ER |  |  |  |  |  |  |  |  |  |
| Negative | 1 |  |  | 1 |  |  | 1 |  |  |
| Positive | 0.886 | 0.338-2.321 | 0.805 | 0.691 | 0.420-1.137 | 0.145 | 1.077 | 0.67-1.732 | 0.759 |
| PR |  |  |  |  |  |  |  |  |  |
| Negative | 1 |  |  | 1 |  |  | 1 |  |  |
| Positive | 0.904 | 0.405-2.014 | 0.804 | 0.683 | 0.461-1.011 | 0.057 | 0.805 | 0.562-1.153 | 0.238 |
| HER2 |  |  |  |  |  |  |  |  |  |
| Negative | 1 |  |  | 1 |  |  | 1 |  |  |
| Positive | 0.831 | 0.411-1.682 | 0.607 | 0.499 | 0.334-0.745 | **0.001** | 0.574 | 0.404-0.815 | **0.002** |
| Neoadjuvant chemotherapy |  |  |  |  |  |  |  |  |  |
| No | 1 |  |  | 1 |  |  | 1 |  |  |
| Yes | 1.52 | 0.755-3.063 | 0.241 | 1.358 | 0.939-1.964 | 0.104 | 1.347 | 0.965-1.879 | 0.08 |
| Endocrine therapy |  |  |  |  |  |  |  |  |  |
| No | 1 |  |  | 1 |  |  | 1 |  |  |
| Yes | 1.004 | 0.385-2.615 | 0.994 | 1.677 | 1.005-2.798 | **0.048** | 0.855 | 0.539-1.354 | 0.503 |
| Targeted therapy |  |  |  |  |  |  |  |  |  |
| No | 1 |  |  | 1 |  |  | 1 |  |  |
| Yes | 5.978 | 2.973-12.021 | **<0.001** | 6.625 | 4.496-9.761 | **<0.001** | 4.671 | 3.293-6.626 | **<0.001** |
| PMRT |  |  |  |  |  |  |  |  |  |
| No | 1 |  |  | 1 |  |  | 1 |  |  |
| Yes | 0.532 | 0.300-0.945 | **0.031** | 0.849 | 0.633-1.137 | 0.272 | 0.76 | 0.586-0.986 | **0.039** |

LRFS, recurrence-free survival; DMFS, distant metastasis-free survival; DFS, disease-free survival; HR, Hazard Ratio; CI, confidence interval; PMRT, postmastectomy radiotherapy; AJCC, American Joint Committee on Cancer; ER, estrogen receptor; PR, progesterone receptor; HER2, human epidermal growth factor receptor 2.
